# Functional Relevance of Coronary Artery Disease by Cardiac Magnetic Resonance and Cardiac Computed Tomography: Myocardial Perfusion and Fractional Flow Reserve

**DOI:** 10.1155/2015/297696

**Published:** 2015-01-27

**Authors:** Gianluca Pontone, Daniele Andreini, Andrea Baggiano, Erika Bertella, Saima Mushtaq, Edoardo Conte, Virginia Beltrama, Andrea Igoren Guaricci, Mauro Pepi

**Affiliations:** ^1^Centro Cardiologico Monzino, IRCCS, Via C. Parea 4, 20138 Milan, Italy; ^2^Department of Cardiovascular Sciences and Community Health, University of Milan, Italy; ^3^Ospedali Riuniti, Department of Cardiology, University of Foggia, Italy

## Abstract

Coronary artery disease (CAD) is one of the leading causes of morbidity and mortality and it is responsible for an increasing resource burden. The identification of patients at high risk for adverse events is crucial to select those who will receive the greatest benefit from revascularization. To this aim, several non-invasive functional imaging modalities are usually used as gatekeeper to invasive coronary angiography, but the diagnostic yield of elective invasive coronary angiography remains unfortunately low. Stress myocardial perfusion imaging by cardiac magnetic resonance (stress-CMR) has emerged as an accurate technique for diagnosis and prognostic stratification of the patients with known or suspected CAD thanks to high spatial and temporal resolution, absence of ionizing radiation, and the multiparametric value including the assessment of cardiac anatomy, function, and viability. On the other side, cardiac computed tomography (CCT) has emerged as unique technique providing coronary arteries anatomy and more recently, due to the introduction of stress-CCT and noninvasive fractional flow reserve (FFR-CT), functional relevance of CAD in a single shot scan. The current review evaluates the technical aspects and clinical experience of stress-CMR and CCT in the evaluation of functional relevance of CAD discussing the strength and weakness of each approach.

## 1. Introduction

Coronary artery disease (CAD) is a major cause of mortality and morbidity in the western countries and its increasing prevalence is responsible for advances in percutaneous and surgical revascularization [1]. The related cost of revascularization procedures has resulted in a great interest of healthcare community regarding appropriateness of this technique and how to select the patients with known or suspected CAD who will receive the greatest benefits from these invasive treatments. Indeed, inappropriate revascularization may generate costs to the healthcare system while appropriate revascularization improves patients' outcome [1]. For this reason, guidelines recommend different diagnostic strategy based on the pretest likelihood of CAD and suggest a conservative observational approach in the case of patients with a very low risk, non-invasive stress testing to detect ischemic burden as gatekeeper to invasive coronary angiography (ICA) in intermediate risk patients, and direct referral for ICA in high-risk patients for CAD. The intermediate risk patients are the most representative population referred to clinical evaluation for suspected or known CAD and the use of imaging tests in this setting has doubled from 2000 to 2006 with $ 14.1 billion in Medicare budget in 2006. Several non-invasive imaging modalities such as exercise electrocardiogram (ECG), stress echocardiography, or nuclear stress tests are suggested as gatekeeper to ICA [1]. However, the diagnostic yield of elective ICA remains low. Patel et al. [2] have showed in a US national register that the prevalence of obstructive coronary artery stenoses in 398978 consecutive patients referred to ICA for suspected CAD was only 38%. The low diagnostic yield of elective ICA occurs despite the fact that 84% of study population undergoing ICA had performed a previous non-invasive diagnostic test. It has also emerged that although non-invasive test was independently related to the presence of obstructive CAD, the additional value of a positive non-invasive stress test to predict obstructive CAD beyond the Framingham risk category and symptom characteristics was limited [2]. However, the evaluation of functional relevance of CAD with noninvasive tests as gatekeeper to ICA remains mandatory. Indeed, the Clinical Outcome Utilizing Revascularization and Aggressive Drug Evaluation trial (COURAGE) [3] and the COURAGE trial nuclear substudy [4] have demonstrated that the event-free survival with coronary revascularization was greater than optimal medical therapy in patients with ≥10% ischemic myocardium at baseline and with a reduction of ischemic myocardium ≥5% after treatment. Stress myocardial perfusion imaging by cardiac magnetic resonance (stress-CMR) has emerged during the past decade as accurate technique for diagnosing and prognostic stratification of the patients with known or suspected CAD thanks to high spatial and temporal resolution, absence of ionizing radiation, and the multiparametric value including the assessment of cardiac anatomy, function, and viability [5]. On the other side, cardiac computed tomography (CCT) has emerged as unique non-invasive technique providing coronary arteries anatomy and more recently as competitive technique able to evaluate the functional relevance of coronary artery stenoses in a single shot scan by using both stress-CCT and fractional flow reserve (FFR-CT) [6]. The current review evaluates the technical aspects and clinical experience of stress-CMR and CCT in the assessment of functional relevance of CAD discussing the strength and weakness of each approach.

## 2. How to Evaluate Functional Relevance of CAD

**Figure 1:**
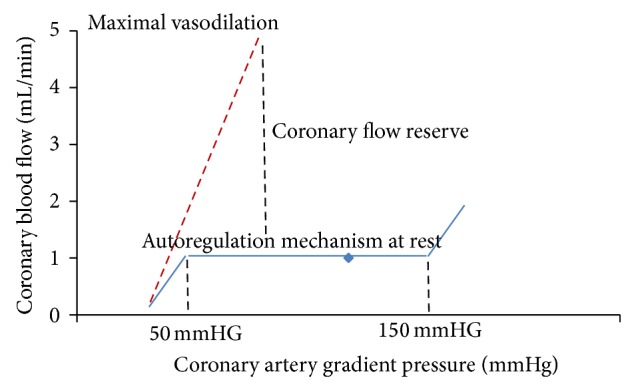
Coronary flow reserve. Relationship between the coronary artery gradient pressure (difference between the epicardial coronary artery and microcirculation pressure) and coronary blood flow at rest condition (blue line) and at maximal vasodilatation (red line). The coronary flow reserve (CRF) is defined as the ratio between the coronary blood flow at maximum vasodilatation and the coronary blood flow at rest condition.

**Figure 2:**
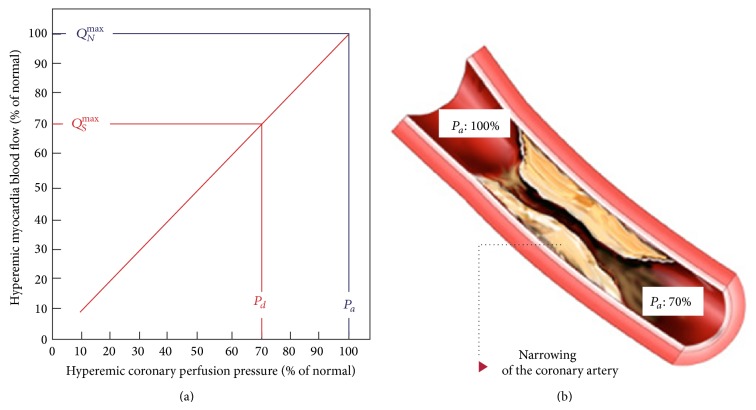
Fractional flow reserve. Left panel shows the relationship between coronary perfusion pressure and myocardial blood flow at maximum hyperemic stimulation. In the absence of coronary artery stenoses, the myocardial blood flow determinated by driving pressure at maximum vasodilatation is 100% (blue line). In case of coronary artery stenoses (right panel) determinating a hyperemic pressure gradient of 30 mmhg (red line) the driving pressure after the stenosis and the consequential myocardial blood flow will be reduced up to 70% corresponding to a FFR value of 0.7. Modified by Pijls et al. [18].

There are two different approaches to evaluate functional relevance of CAD: the assessment of myocardial perfusion under stress and the measurement of FFR. The rationale for stress myocardial perfusion imaging is based on the concept of coronary flow reserve [7] that is briefly described in Figure 1. The cardiac metabolism is mainly aerobic and it is sustained by a coronary blood flow of about 0.8–1 mL/gr/min that occurs in diastolic phase and it is driven by gradient between the diastolic blood pressure and the end-diastolic left ventricle pressure [7]. At rest condition, the autoregulation mechanism by adjusting coronary microvascular resistance maintains coronary blood flow constant in a wide range of coronary perfusion gradient pressure and the heart utilizes the maximum oxygen extraction corresponding to 80% of the oxygen available in the blood pool. Therefore, in the conditions in which the oxygen demand is increased, a maximal coronary artery vasodilatation occurs to reach a direct relationship between coronary artery gradient pressure and coronary artery blood flow. The coronary artery flow reserve (CFR) is the ratio between the coronary artery flow at maximum vasodilatation and the coronary blood flow at rest. Usually, in the absence of epicardial coronary artery disease, CRF increases by factor 5 or more while in presence of 50% epicardial coronary artery lumen reduction CRF decreases up to be abnormal at rest in the presence of high degree of stenoses (>85%) [8, 9]. The maximal vasodilatation can be brought with pharmacologic stress by using three different drug stressors such as adenosine, dipyridamole, or regadenoson that are briefly described in Table 1. Both adenosine and dipyridamole are direct and indirect not-selective adenosine receptor agonists, respectively, providing up to fourfold increase of coronary flow. In the presence of epicardial coronary stenoses both drug stressors act on normal vessels but have no effects on stenotic vessels producing ischemia based on “steal phenomenon” [10]. More recently, regadenoson, a selective A_2A_ receptor agonist, has been approved for pharmacological stress test with the great advantage of having minor side effects as compared to adenosine and dipyridamole [11]. Of note, the myocardial perfusion can be evaluated by dobutamine as well. However, dobutamine induces ischemia by improving heart rate rather than “steal phenomen”. Therefore, the perfusion defect detected by dobutamine is an indirect effect of mismatch between the mycoradial perfusion and myocardial oxygen consumption due to increase of heart rate. The myocardial ischemia induced by “steal phenomenon” is the base of perfusion and wall motion abnormalities detection during stress-CMR and stress-CCT. Unfortunately, the CFR is an expression of a pressure gradient between epicardial coronary artery and microcirculation and therefore it is reduced in case of collaterals or microcirculation disease even in the absence of epicardial stenosis [12]. For this reason, these stress perfusion tests are not able to distinguish between the two entities. To this regard, the invasive FFR has been introduced to overcome these limitations. FFR is performed by dedicated solid-state sensor mounted on a floppy-tipped guidewire. It measures the intracoronary pressure before and after a specific coronary lesion in the presence of hyperaemic stimuli by adenosine reaching a direct relationship between pressure and flow. Therefore, FFR can be expressed as the ratio of maximum blood flow after coronary artery stenoses to maximum blood flow. Coronary artery lesions with FFR ≤ 0.80 have been proved to receive benefits from revascularization while, in a setting of a stenosis with a FFR > 0.80, the patient can be safely deferred to optimal medical treatment [13, 14]. More important, unlike the ischemia stressors induced, FFR is not influenced by systemic hemodynamic [15, 16], it takes into account the contribution of collaterals [17, 18], it specifically relates to the severity of the stenoses and to the mass of tissue to be perfused [19], and it reaches a per-lesion accuracy rather than per-myocardial territory with a very high spatial resolution [12]. The main principles of FFR are described in Figure 2. In conclusion, it is of paramount importance to determine if a coronary stenosis is associated with reversible ischemia for decision making of treatment, and myocardial perfusion under stress or FFR are two sides of same coin.

### 2.1. Functional Relevance of CAD by CMR 

#### 2.1.1. Principles of Stress Cardiac Magnetic Resonance Protocol

**Figure 3:**
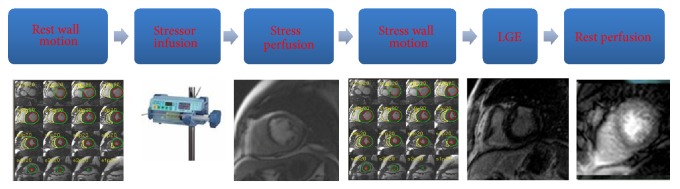
Stress cardiac magnetic resonance protocol. A typical stress cardiac magnetic resonance protocol is described here. For the description see throughout the paper. LGE: late gadolinium enhancement.

Despite the fact that the technical aspects of stress-CMR are beyond the aim of this paper, a summary of the most common protocols used is described below and illustrated in Figure 3 according to the recommendations of the Society of Cardiovascular Magnetic Resonance [20]. After steady-state free precession cine acquisitions have been acquired at rest during held expiration in multiple short and long axes, coronary vasodilatation can be induced with drug infusion and then first-pass perfusion technique using saturation-prepared T1-weighted fast gradient-echo sequence with simultaneous gadolinium contrast agent injection. In case of use of dipyridamole as stressor, due to the longer half-life as compared to adenosine, left ventricle kinesis under stress can be evaluated by further steady-state free precession cine acquisitions with the same geometry used at rest. Theophylline is intravenously injected to null the effect of dipyridamole at the end of stress test. Ten minutes after contrast injection, breath-hold contrast-enhanced segmented T1-weighted inversion-recovery gradient-echo sequences are acquired with the same prescriptions for cine images to detect late gadolinium enhancement (LGE). At the end of exam, a further first-pass perfusion technique is performed to provide myocardial perfusion at rest.

#### 2.1.2. Principles of Evaluation of Reversible Ischemia

**Figure 4:**
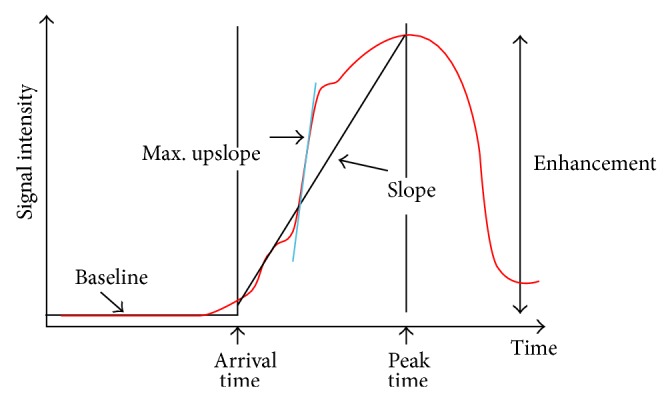
Time course of gadolinium myocardium enhancement. The time attenuation curve within a region of interest is obtained by fitting several sampling of myocardial signal intensity over time during the first pass imaging. For each curve arrival time, peak time and slope can be measured and compared between stress and rest condition. Modified by Patel et al. [21].

The clinically predominant mode of reading and interpreting myocardial perfusion studies is based on visual approach. Beyond the usual parameters estimated by CMR such as end diastolic and end systolic left and right ventricle volume, left ventricle mass, and left and right ejection fraction, a reversible perfusion defect is defined as persistent delay of enhancement during first pass of contrast agent for >3 heartbeats after maximum signal intensity in the cavity of the left ventricle that does not correspond to perfusion defect at rest. Similarly, each myocardial segment is classified as normal, hypokinetic, akinetic, or dyskinetic. Accordingly, each stress-CMR can be classifiedas normal for reversible ischemia (no evidence of ischemia due to the absence of stress perfusion defect in at least 1 myocardial segment free from LGE), positive for reversible myocardial perfusion defect alone (evidence of stress perfusion defect in at least 1 myocardial segment without corresponding LGE), or positive for both perfusion and wall motion abnormalities (WMA) (evidence of stress perfusion defect in at least 1 myocardial segment without corresponding LGE plus stress WMA as compared to rest condition). A quantitative analysis of the myocardial perfusion is feasible as well. The epicardial and endocardial borders of the left ventricle wall have to be detected for each image frame of perfusion study to derive parameters value of time course of contrast agent as showed in Figure 4. For each curve, a time to arrival of contrast agent, a time to peak of signal intensity, or the slope of curve can be calculated and compared between stress and rest. Despite the fact that the quantitative approach has been proved more robust to discriminate between 1-vessel and 3-vessel disease, it is extremely time consuming and therefore not generally used in clinical practice [21]. Several artifacts can occur during stress-CMR. Dark subendocardial rim artifacts are the most common and may be confused with myocardial perfusion defects. They typically appear as dark lines at the border of blood flow and myocardium. Factors that may contribute to the production of dark rim artifacts include partial-volume averaging, gadolinium-induced magnetic susceptibility, myocardial motion, and undersampling from low spatial resolution, either alone or in combination. Regardless of their cause, the artifacts are mitigated by using parallel acquisition schemes, by reaching greater SNR with 3.0-T magnets, or by using lower concentrations of gadolinium leading to less severe magnetic susceptibility effects. Aliasing artifacts are common as well in first-pass perfusion imaging especially when parallel imaging techniques are used and they could be mitigated by selection of a sufficiently large field of view.

#### 2.1.3. Stress-CMR for Diagnosis of CAD

**Figure 5:**
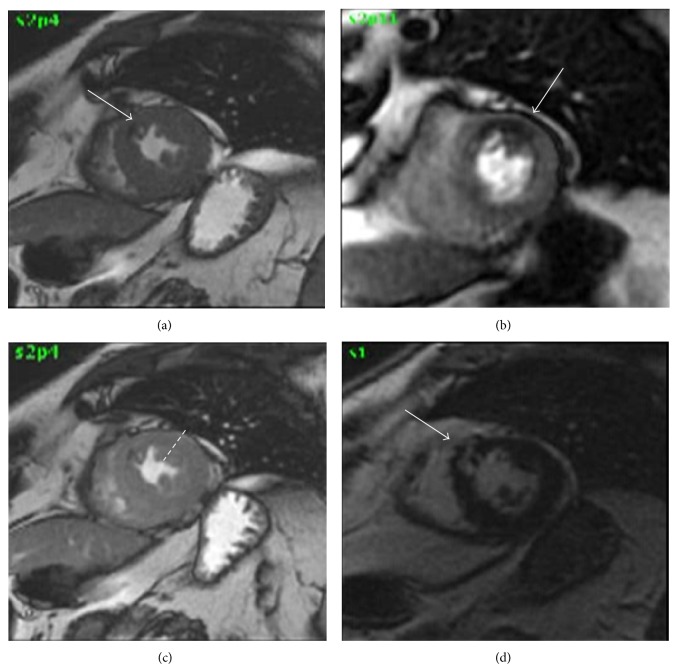
Clinical case. Dipyridamole stress-CMR in a 62-year-old man with familiar history of CAD and hypertension, referred to chest pain. Cine images at rest (Panel (a)) showed akinesis of anterior interventricular septum (arrow). Under stress, first pass perfusion images showed a large perfusion defect in anterior wall of left ventricle (Panel (b), arrow) without matched wall motion abnormalities (Panel (c), arrow). Late gadolinium enhancement images show an unknown transmural myocardial infarction of anterior interventricular septum (Panel (d), arrow).

A high number of single and multicenter studies have proved the excellent sensitivity and specificity of stress-CMR for diagnosis of CAD [22–25] and are briefly described in Table 2. In a meta-analysis by Nandalur et al. [22] involving 1183 patients, perfusion CMR had a sensitivity of 91% and a specificity of 81% and a stress-induced WMA demonstrated a sensitivity of 83% and specificity of 86% for diagnosis of CAD in a per-patient analysis, respectively. Moreover, several papers have compared stress-CMR versus single photon emission computed tomography (SPECT) in terms of diagnostic accuracy. Schwitter et al. [23] compared stress-CMR versus SPECT in 234 patients by using ICA as reference showing a better performance of stress-CMR versus SPECT with an area under the curve (AUC) of 0.86 ± 0.06 versus 0.67 ± 0.05 (*P*: 0.013), respectively. It is important to note that in this study, gated-SPECT, that is considered now the standard technique for stress nuclear tests, was available in approximately half of patients. Moreover, the population evaluated in MR-IMPACT I was at high risk for CAD that is not the typical population referred to noninvasive stress tests in clinical practice. In MR-IMPACT II trial [25] 533 patients were enrolled in 33 centres and evaluated by stress-CMR and gated-SPECT before ICA. The study population was at intermediate risk of CAD as proved by prevalence of obstructive coronary stenoses. No differences were found between the stress-CMR and SPECT in terms of percentage of not-evaluable tests (5.6% versus 3.7%, resp.; *P*: 0.21) while stress-CMR showed a higher sensitivity score (0.67 versus 0.59, resp.; *P*: 0.024) but a lower specificity score (0.61 versus 0.72, resp.; *P*: 0.038). In the larger multicenter trial CE-MARC [24], stress-CMR and SPECT showed sensitivity, specificity, positive predictive value, and negative predictive value of 86%, 83%, 77%, and 90% and 66%, 82%, 71%, and 79%, respectively. The sensitivity and negative predictive value of stress-CMR and SPECT differed significantly (*P* < 0.0001 for both) but specificity and positive predictive value did not. Moreover, stress-CMR showed a higher AUC as compared to SPECT (0.89 versus 0.79; *P* < 0.0001) regardless of the threshold used to define the presence of obstructive CAD (50% or 70% of coronary artery stenoses) and regardless of the presence of one or multiple vessels disease. Importantly, in CE-MARC trial a multiparametric protocol has been used including wall motion at rest by balanced steady-state free precession cine imaging, stress and rest perfusion by T1-weighted saturation recovery, evaluation of coronary artery stenoses by 3D coronary magnetic resonance angiography, and LGE by T1-weighted segmented inversion-recovery gradient-echo pulse sequence. A positive stress-CMR was defined as any evidence of regional wall motion abnormality and/or perfusion defect at stress and/or the presence of obstructive coronary artery stenoses and/or any scar. Of note in a gender-based subanalysis of CE-MARC trial [26] stress-CMR has greater sensitivity than SPECT in both genders and, unlike SPECT, there are no significant gender differences in the diagnostic performance. In Figure 5, a clinical case of a 62-year-old man referred to dipyridamole stress-CMR for exertional chest pain is reported.

#### 2.1.4. Stress-CMR for Prognostic Stratification of CAD

Over the past several years, multiple studies have been published regarding stress-CMR assessment of prognosis. In a recent meta-analysis, Lipinski et al. [27] showed in 11636 patients that the combined outcome annualized event rates were 0.8% for negative study and 4.9% for positive study with the evidence of LGE significantly associated with worse prognosis as well. Macwar et al. [28] found an annual event rate for hard events of 0.6%, 1.7%, and 1.5% in normal, positive for LGE, and positive for reversible perfusion defect adenosine stress-CMR, respectively, in 564 patients symptomatic for chest pain without previous history of revascularization. Similarly, Buckert et al. [29] showed a hazard ratio of 3.2 associated with reversible perfusion defect in a larger population (1152 patients) in a long-term follow-up (4.2 years). These data support consistent and robust prognostic stratification by adenosine stress-CMR and it seems that this robustness is preserved regardless of patient's gender [30]. Regarding dobutamine studies, Kelle et al. [31] showed that, in a large cohort (1369 patients) evaluated with dobutamine stress-CMR, the annual cardiac event rate of a negative stress test was 1.1%, while the hazard ratio associated with a positive dobutamine stress test was 3.3. Similarly, Wallace et al. [32] found that the presence of inducible AWM is associated with a hazard ratio of 2.7 for future hard cardiac events in 221 consecutive women. Only few studies have tested the usefulness of dipyridamole stress-CMR for predicting spontaneous clinical events [33, 34]. Bodi et al. [33] found that the prognostic value of perfusion deficit was weaker than AWM under stress suggesting that kinesis evaluation is desirable beyond the perfusion. The latter point was evaluated in a following paper from the same authors [34] in 601 consecutive patients with a mean follow-up of 640 days with an annual hard event rate of 2.9%, 11.7%, and 14.1% in the three categories described above, respectively. This progressive increase of hard event rate in these three categories finds an explanation in the evidence that the extent of the perfusion defect is larger in patients with concomitant AWM [34, 35].

#### 2.1.5. Future Perspectives

Three main fields of investigation are developing in the detection of CAD by CMR. First, whole heart stress-CMR perfusion techniques have been developed in order to permit quantification of ischemic tissue volume [36]. This approach proved to be highly diagnostic for the detection of CAD based on invasive FFR, showing similar sensitivity but improved specificity (90 versus 92% and 82 versus 74%, resp.) as compared to previously published data. Jogiya et al. [37] demonstrated that whole heart myocardial perfusion CMR accurately detects functionally significant coronary artery disease highlighting the concept that diagnostic accuracy was equally high in patients with single-vessel and multivessel coronary artery disease and that the amount of myocardial ischemic burden gradually increased with more proximal anatomical localization of coronary lesions. This method may be considered a noninvasive approach to stratify patients before coronary angiography to identify that cut-off of 10% of myocardial ischemic burden that is actually accepted as threshold for indication of interventional revascularization. Second, the “blood oxygen level-dependent” (BOLD) stress-CMR [38] has become a promising investigative method. The rationale of this promising method is that oxyhemoglobin is slightly diamagnetic and deoxyhemoglobin paramagnetic resulting in the loss of T2^*^ and T2 signal. Therefore, BOLD CMR uses an endogenous contrast without additional use of an exogenous contrast. Using a T2-prepared steady-state free precession, BOLD stress-CMR sequences can be used to image changes of myocardial oxygenation induced by drug stressors as an indicator of myocardial ischemia. Myocardial perfusion assessed by the change in myocardial oxygenation with those new sequences has been correlated with quantitative coronary angiography and later with fractional flow reserve to better characterize the clinical significance of coronary stenosis [38]. Third future perspective is to visualize the atherosclerotic plaque of coronary arteries. Early reports demonstrated the principal feasibility of the contrast enhancement magnetic resonance coronary angiography (CEMRA) to visualize the coronary artery stenoses noninvasively showing some limitations both in sensitivity and in specificity as compared to ICA [39, 40]. A new family of contrast agents that may play an important role in investigating molecular and cellular targets associated with atherosclerotic plaque development has been recently shown [41, 42]. A prospective multicenter trial [43] evaluated the diagnostic ability of navigator-corrected SSFP whole-heart MRCA sequences to detect significant coronary artery stenosis (≥50% reduction in diameter) with ultrasmall superparamagnetic iron oxide nanoparticles showing a sensitivity and a specificity of 88% and 72% with a negative predictive value of 88% in a patient-based analysis as compared to ICA.

### 2.2. Functional Relevance of CAD by CCT

The advancement of CCT has enabled the noninvasive evaluation of coronary artery stenosis in several clinical scenarios [44–48] with a low radiation exposure [49–51]. Despite high negative predictive value, factors such as high heart rate, arrhythmia, obesity, and high coronary calcium burden continue to limit the overall evaluability and positive predictive value of CCT [52, 53]. Moreover, obstructive CAD identified by CCT is not a robust predictor of functional relevance of stenoses [54]. So, the combination of overestimation of CAD and the absence of functional information could be responsible for a false positive rate up to 35% of patients that is common event in experienced centers [55]. Thus, a combined anatomic and functional assessment of CAD is desirable to improve the yield of ICA [56]. This aim can be reached by stress-CCT and FFR-CT. In this section, we will briefly summarize the technical bases and preliminary clinical findings of these emerging novel techniques.

#### 2.2.1. Perfusion Stress Cardiac Computed Tomography

**Figure 6:**
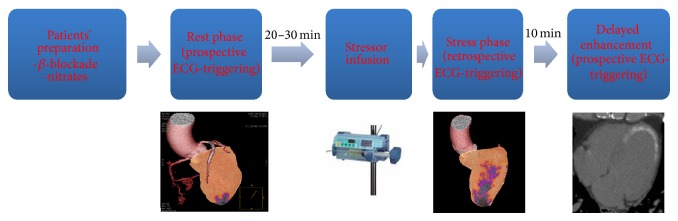
Scan protocol of stress cardiac computed tomography.

**Figure 7:**
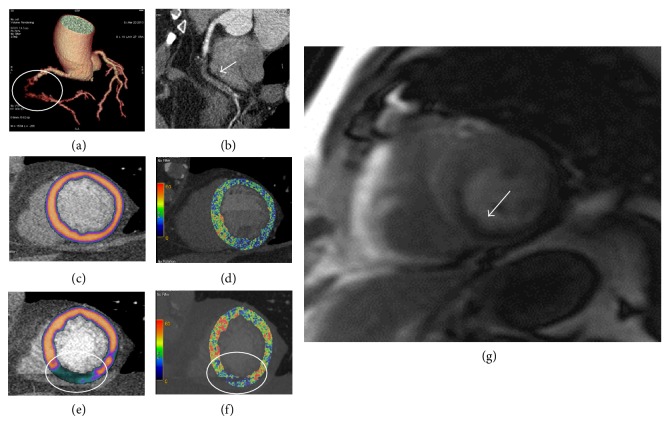
Clinical case of a 61-year-old man referred for suspected coronary artery disease. To rule out the presence of significant coronary artery disease and ischemia, a rest-stress dual energy CCT has been performed. The exam showed a chronic total occlusion of the right coronary artery (Panel (a) circle, Panel (b) arrow) due to a noncalcified plaque. The myocardial perfusion (Panel (c)) and iodine map (Panel (d)) at rest do not show significant perfusion defect. Under stress condition (i.v. adenosine injection), dual energy computed tomography showed a large perfusion defect in inferior wall of left ventricle (Panel (e) and (f), circle) with good matching as compared to stress cardiac magnetic resonance (Panel (g), arrow). CCT: cardiac computed tomography.

Investigation in the field of stress-CCT started 2 decades ago [57, 58]. Unfortunately, the clinical use of this technique has remained restricted due to several technical limitations such as the limited temporal resolution, spatial and contrast resolution, and the *z*-axis coverage. Indeed, during a stress-CCT, the contrast agent arrives to myocardial wall and it attenuates X-ray based on its concentration. Thus, area with perfusion defect is simply detectable as region with hypoattenuation. However, the highest concentration of iodine in the myocardium is reached in 1 minute after injection with a very fast washout [59]. To imagine this very rapid phenomenon, a high temporal resolution is mandatory. Some strategies have been used to improve temporal resolution of CCT [60]. The first strategy is to increase gantry rotation time. Indeed, in order to accurately reconstruct an image, the projection data are acquired within an angular range of 180° plus a 60° fan angle with a minimal data acquisition angular range of 240°. This fact determines that the typical temporal resolution of CCT is about 50–60% of the gantry rotation time [61]. Accordingly, the highest maximum temporal resolution achievable for single source scanner is 135 msec that is far from the desirable temporal resolution reached by ICA that is around 20–30 msec. The introduction of dual source CT (DSCT) has partially overcome this limitation. This system combines 2 arrays consisting of 1 tube and 1 detector each, arranged within the same gantry at a 90° offset, so that one-quarter rotation is sufficient to sample X-ray transmission data over 180° of projections. With a gantry rotation time of 330 ms, the system could achieve a temporal resolution of 83 ms that is significantly higher than single source CCT [62]. More recently, intracycle motion correction algorithm that allows a compensation of coronary motion by using multiphasic analysis of coronary arteries within a single cardiac cycle has been developed for single source scanner reaching a temporal resolution of 25 msec [63, 64]. However, a high temporal resolution needs to be matched with a very fast scan time. There are two strategies to improve the scan time: a higher craniocaudal coverage by increasing the number of slices or a higher pitch or the combination of the two strategies. Craniocaudal coverage of 64-slice CCT coronary angiography is typically less than 40 mm, giving limited coverage width. The development of wide area detector CCT [65] enabled greater coverage per gantry rotation and the extension from 64-slice MDCT to 256-detector row or 320-detector row system has enabled whole heart coverage. The 256-CCT has 912 (transverse) × 256 (craniocaudal) elements, each approximately 0.5 × 0.5 mm at the center of rotation with craniocaudal coverage of 128 mm per rotation. The 320-CCT system uses a detector element consisting of 320 × 0.5 mm detector and provides 160 mm of coverage in the *z*-direction [66]. The scan time can be reduced by increasing the pitch, as well. In 2008, a high pitch scanner has been introduced. It rotates with a gantry rotation time of 280 milliseconds, a wider detector with a pitch up to 3.2 that allows an overall scan time of about 0.27 seconds to cover the entire heart. This scan mode, known as “Flash CT,” enables complete image acquisition within one cardiac cycle so that the X-ray tube and detector rotate around the patient without overlap and with very short scan time [67]. After temporal resolution, a second challenge in stress-CCT is to improve the spatial and contrast resolution in the myocardial wall. Indeed, the difference in terms of contrast attenuation between normal and hypoperfused myocardial regions ranges in the order of 50 HU [68]. To highlight the small differences in contrast attenuation between normal and hypoperfused myocardium, it is suggested to use low tube voltage (100 KVp) to increase the photoelectric effect and to decrease the Compton scattering associated with low tube voltage [69]. However, the low tube voltage increases the image noise and the use of adaptive iterative reconstruction algorithm rather than the filtered back projection algorithm is recommended to limit the image noise increase when low tube voltage is employed [50]. Despite the use of low tube voltage and iterative reconstruction algorithm, the beam hardening artefacts continue to be an issue of concern during a stress-CCT because they can mimic a false perfusion defect. These artifacts are due to the polychromatic nature of X-rays and the energy dependency of X-ray attenuation phenomenon [6]. Indeed, X-ray photons with lower energies are preferentially attenuated and this inconsistency of X-ray between different views results in misregistration of X-ray attenuation producing false perfusion defect [6]. Recently the dual energy computed tomography (DECT) has been introduced to overcome this limitation [70]. This technique simultaneously acquires 2 sets of projections using 2 different X-ray energy spectra. In this way, DECT seems to be more effective for correcting beam hardening artifacts due to the ability to reconstruct monochromatic CCT images [71]. So far, based on technology available, two main kinds of stress-CT can be performed. Static stress-CCT imaging is usually preferred for scanner with low temporal resolution and long scan time in which the myocardial perfusion is reached from a single data sample acquired in arterial phase timing. On the other side, the dynamic stress-CCT imaging, usually performed with scanner with high temporal resolution and low scan time, is obtained from multiple samples of myocardial attenuation at sequential time points after contrast injection with a method similarly to the stress-CMR previously described. For both approaches, the adenosine infusion is usually used as stressor with the same protocol described for stress-CMR. In Figure 6, the most common protocol used in clinical practice is briefly described. Up to now, a few single-centre studies [72–83] are available regarding the diagnostic accuracy of stress-CCT and they are summarized in Table 3 with a mean effective radiation dose of rest plus stress scan of about 11 mSv that is comparable with the mean radiation exposure usually associated with SPECT [51]. More recently, a multicenter trial CORE 320 [84] has evaluated the diagnostic accuracy of stress-CCT as compared to the combination of SPECT plus ICA. The results of this trial showed in a patient-based model AUC of 0.87 for integrated CCT and perfusion as compared to the algorithm SPECT plus ICA.  Figure 7 showed one case of stress-CCT with single source dual energy technique.

#### 2.2.2. Fractional Flow Reserve by Cardiac Computed Tomography (FFR-CT)

**Figure 8:**
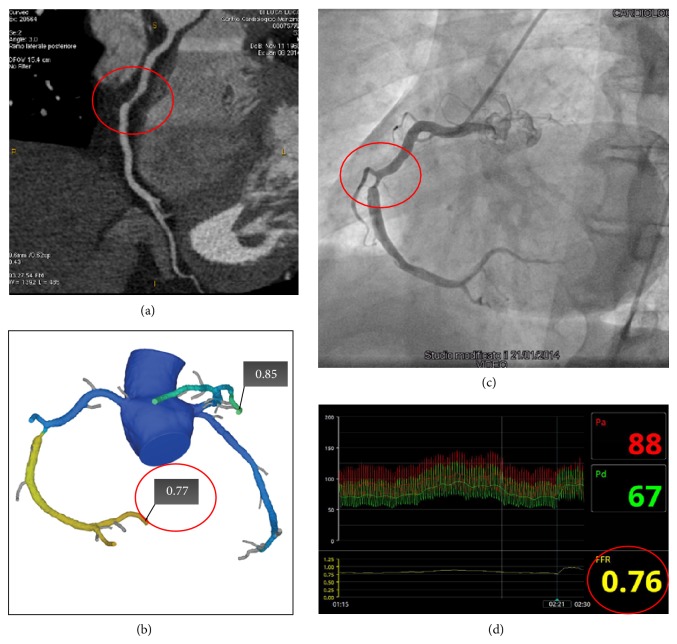
Clinical case of an intermediate risk patient symptomatic for chest pain. Panel (a) showed a multiplanar reconstruction of CCT showing an obstructive stenosis of the middle right coronary artery (red circle) and computed fractional flow reserve (FFR-CT) value of 0.77, indicating vessel ischemia (Panel (b)). Invasive coronary angiography confirmed the obstructive stenosis of the middle portion of the right coronary artery (Panel (c), red circle) and measured fractional flow reserve (FFR) values of 0.76 (Panel (d), red circle). CCT: cardiac computed tomography.

In the “era” of FAME trial an emerging interest has been increased on the possibility to measure in a noninvasive setting the FFR. To this regard, software to determine FFR which computes the hemodynamic significance of CAD from CCT dataset (FFR-CT) using computational fluid dynamics under rest and simulated maximal coronary hyperemic conditions has been recently developed [85]. Although the description of technical aspects of this method is beyond the aim of this paper, the computation of FFR-CT can be summarized as an integration of anatomic model of coronary arteries derived by CCT plus a mathematical model of coronary physiology including numerical solution of the laws of physics governing fluid dynamics [85]. In other words, the combination of high-resolution anatomic definition of CAD via CCT with FFR-CT in a single test without the use of stressors provides a noninvasive anatomic and functional assessment of CAD in one-shot scan without the need of additional functional test in case of obstructive CAD at CCT. Results from 3 prospective multicenter trials have validated the accuracy of FFR-CT as compared to invasive FFR [86–88]. Koo et al. compared in DISCOVER-FLOW trial the FFR-CT versus invasive FFR in 103 consecutive patients in a vessel-based model showing diagnostic accuracy, sensitivity, specificity, positive predictive value, and negative predictive value of 84%, 88%, 82%, 74%, and 92%, respectively. The FFR-CT increased the AUC from 0.75 for CCT alone up to 0.91 (*P*: 0.001) and showed a good correlation with invasive FFR (0.71, *P* < 0.001). The main limitation of DISCOVER-FLOW is that it was powered to evaluate the diagnostic accuracy in a per-vessel model rather than per-patient model. The latter point was evaluated in DE-FACTO trial [87] where, in larger sample size of 252 patients, the FFR-CT showed diagnostic accuracy, sensitivity, specificity, positive predictive value, and negative predictive value of 73%, 90%, 54%, 67%, and 84%, respectively, improving the AUC from 0.68 to 0.81 when compared with CCT alone without functional evaluation. More recently, the NXT trial [88] has demonstrated per-patient sensitivity and specificity of 86% and 79%, respectively, with AUC of 0.9 as compared to invasive FFR. Of note, in patents with intermediate stenoses (the most common setting of patients evaluated with noninvasive stress test) the diagnostic performance of FFR-CT remained unchanged. Differently for the two previous studies, the last generation of FFR-CT software has been used, nitrates was employed in 99% of study population against only 75% of DEFACTO study population, and, last but not least, an intermediate risk population was included rather than high-risk patients such as the two previous studies. The main limitation of FFR-CT remains still the not-negligible rate of not-evaluable patients that reaches the 13% in the NXT-trial due to the poor image quality. So far, the clinical validation of FFR-CT opens the issue if the use of this software integrated to CCT dataset is reasonable in terms of cost-effectiveness in intermediate risk patients by “one-stop shop” offering coronary anatomy and functional relevance of CAD. The final answer to this crucial question will arrive with the ongoing PLATFORM trial (Prospective LongitudinAl Trial of FFRct: Outcome and Resource IMpacts) that has the aim to compare the rate of ICA documenting nonobstructive CAD, clinical outcomes, quality of life, and resource utilization following standard practice versus incorporating FFR-CT as the preferred test to guide further noninvasive or invasive management and medical treatment of patients.  Figure 8 shows a clinical case of patient in which FFR-CT has been performed and compared to invasive FFR.

## 3. Conclusions

The world of cardiac imaging in the field of CAD is proposing an increasing number of techniques with the aim to rule out the presence of CAD and to identify the high-risk patients who will benefit from expensive invasive procedures. There is robust evidence that, to reach this aim, both coronary anatomy and function need to be evaluated. How to obtain combined anatomic and functional noninvasive imaging by using one or multiple imaging modalities still has not been demonstrated and it will be issue of debate for the next years ensuring that “exciting times are ahead of cardiac imagers.”

## Figures and Tables

**Table 1:** Summary of the most common drug stressors used for the evaluation of myocardial perfusion.

Drug stressors	Action	Half-life	Side effects	Cost
Adenosine	Coronary vasodilatation induced by not-selective A1 adenosine-receptors stimulation (increasing cellular cAMP levels)	10 seconds	Facial flushing Diaphoresis nausea asthma Bradyarrhythmias	↑↑↑

Dipyridamole	Coronary vasodilatation induced by inhibition of the phosphodiesterase enzymes that normally break down cAMP (increasing cellular cAMP levels)	25 minutes	Facial flushing Diaphoresis nausea asthma Bradyarrhythmias	↑

Regadenoson	Coronary vasodilatation induced by selective A2A adenosine-receptors stimulation	2-3 minutes	Headache, dizziness, nausea, stomach discomfort, decreased sense of taste, mild chest discomfort, or warmth, redness, or tingly feeling under your skin	↑↑

**Table 2:** Characteristics of a selected list of studies of the diagnostic performance of stress myocardial perfusion cardiac magnetic resonance.

Author	Reference	*N*	Sensitivity	Specificity
Nandalur et al. [22]	Stress-CMR versus QCA (meta-analysis)	1183	91%	81%
Schwitter et al. [23]	Stress-CMR and SPECT versus QCA	234	67%	85%
Greenwood et al. [24]	Stress-CMR and SPECT versus QCA	752	86%	83%
Schwitter et al. [25]	Stress-CMR and SPECT versus QCA	533	75%	59%
Greenwood et al. [26]	Stress-CMR and SPECT versus QCA	235	88%	83%

CMR: cardiac magnetic resonance; QCA: quantitative coronary angiography; SPECT: single photon emission computed tomography.

**Table 3:** Characteristics of a selected list of studies of the diagnostic performance of stress myocardial perfusion cardiac computed tomography imaging.

Author	Reference	*N*	Effective radiation dose (mSv)	Sensitivity	Specificity
George et al. [73]	Stress-CCT versus QCA + SPECT	27	16.8	86%	92%
Blankstein et al. [74]	Stress-CCT and SPECT versus QCA	33	12.7	92%	67%
Rocha-Filho et al. [75]	Stress-CCT versus QCA	34	11.8	96%	100%
Cury et al. [77]	Stress-CCT and SPECT versus QCA	36	14.7	94%	75%
Ho et al. [78]	Stress-CCT versus QCA + SPECT	35	18.2	95%	65%
Bamberg et al. [81]	Stress-CCT versus invasive FFR	33	13.1	95%	64%
Feutchtner et al. [82]	Stress-CCT versus stress-CMR	30	2.5	96%	88%

CCT: cardiac computed tomography; CMR: cardiac magnetic resonance; FFR: fractional flow reserve; QCA: quantitative coronary angiography; SPECT: single photon emission computed tomography.
